# Self-reported hearing loss questions provide a good measure for genetic studies: a polygenic risk score analysis from UK Biobank

**DOI:** 10.1038/s41431-020-0603-2

**Published:** 2020-03-20

**Authors:** Stacey S. Cherny, Gregory Livshits, Helena R. R. Wells, Maxim B. Freidin, Ida Malkin, Sally J. Dawson, Frances M. K. Williams

**Affiliations:** 1 Department of Anatomy and Anthropology, Sackler Faculty of Medicine, Tel Aviv University, Tel Aviv, Israel; 2 Department of Epidemiology and Preventive Medicine, Sackler Faculty of Medicine, Tel Aviv University, Tel Aviv, Israel; 3 Department of Twin Research and Genetic Epidemiology, School of Life Course Science, King’s College London, London, UK; 4 Adelson School of Medicine, Ariel University, Ariel, Israel; 5 Centre for Auditory Research, UCL Ear Institute, University College London, London, UK

**Keywords:** Population screening, Preventive medicine, Genetic testing

## Abstract

Age-related hearing impairment (ARHI) is very common in older adults and has major impact on quality of life. The heritability of ARHI has been estimated to be around 50%. The present study aimed to estimate heritability and environmental contributions to liability of ARHI and the extent to which a polygenic risk score (PRS) derived from a recent genome-wide association study of questionnaire items regarding hearing loss using the UK Biobank is predictive of hearing loss in other samples. We examined (1) a sample from TwinsUK who have had hearing ability measured by pure-tone audiogram and the speech-to-noise ratio test as well as questionnaire measures that are comparable with the UK Biobank questionnaire items and (2) European and non-European samples from the UK Biobank which were not part of the original GWAS. Results indicated that the questionnaire items were over 50% heritable in TwinsUK and comparable with the objective hearing measures. In addition, we found very high genetic correlation (0.30–0.84) between the questionnaire responses and objective hearing measures in the TwinsUK sample. Finally, PRS computed from weighted UK Biobank GWAS results were predictive of both questionnaire and objective measures of hearing loss in the TwinsUK sample, as well as questionnaire-measured hearing loss in Europeans but not non-European subpopulations. These results demonstrate the utility of questionnaire-based methods in genetic association studies of hearing loss in adults and highlight the differences in genetic predisposition to ARHI by ethnic background.

## Introduction

Age-related hearing impairment (ARHI) is prevalent in older adults, affecting at least 60% of people by the time they reach 71–80 years of age [1]. The number of people with ARHI will necessarily increase as humans live longer and a greater proportion of the population is older [2]. Hearing loss does not just impact communication; it is associated with loneliness and depression, cognitive decline, and dementia, as well as reduced physical well-being [3–6]. A recent review estimated excess direct medical costs as a result of hearing loss to be between US$3.3 billion and US$12.8 billion in the United States, with the economic costs due to lost productivity ranging from US$1.8 billion to as much as US$194 billion [7]. There is currently no effective drug treatment for hearing loss. Hearing aids (HAIDs) (average cost £2300/pair) are the most commonly prescribed ameliorative therapy but uptake is low, and among those who obtain HAIDs, a high proportion do not use them or are dissatisfied with them. Prevention of hearing loss is therefore of compelling necessity, and genetic studies will help us understand how and why people lose their hearing ability and will inform prevention strategies. Despite its high prevalence and substantial genetic variance [8], our understanding of the precise genes responsible for ARHI has been limited.

ARHI results from the cumulative effects of individual environmental and lifestyle-related experiences that interact with genetic factors to make some people more susceptible to hearing loss than others. Variable susceptibility results in wide variation in the severity of ARHI, so that some people will experience significant, disabling levels of hearing loss while others retain good hearing ability well into old age [9]. Genetic susceptibility is a key factor that explains this variation with estimates of hearing heritability to be between 46 and 74% [8, 10–13]. Earlier twin studies examining hearing ability by age found heritability to decline from 100% in the 35–45 years old group to 47% in the over 65 group [14]. Another study of twins aged 70 and older found heritability to be 40%, consistent with this earlier study [15].

Two novel loci for ARHI have recently been identified in an analysis of the non-Hispanic whites in a large (6527 cases and 45,882 controls) cohort, Genetic Epidemiology Research on Adult Health and Aging (GERA) [16]. These loci replicated in both the remaining other ethnic group samples of GERA and the UK Biobank (UKB) cohort, at the time comprising just over 100,000 samples. Most recently, the full UKB dataset had become available (~500,000 samples) and we performed two large genome-wide association studies (GWAS) for two questionnaire responses assessing ARHI on the white British portion of the sample of just over 250,000 individuals, derived from both principal component (PC) analysis and self-declared ethnicity [17]. The questionnaire items used were ‘Do you use a hearing aid most of the time?’ (HAID) and, combined responses to ‘Do you have any difficulty with your hearing?’ and ‘Do you find it difficult to follow a conversation if there is background noise (such as TV, radio, children playing)?’ (hearing difficulties; HD). GWAS yielded 41 statistically significant independent associated genomic regions (2080 single-nucleotide polymorphisms; SNPs) for HD and 7 (240 SNPs) for HAID. Four of the HD variants lie in exons and code missense variants in *EYA4*, *CDH23*, *KLHDC7B*, and *TRIOBP* genes, while 25 lie within introns. Loci common to both traits were *NID2*, *ARHGEF28*, and *EYA4*. *EYA4* variants have been implicated previously in other forms of hearing loss [18], but *NID2* and *ARHGEF28* were novel associations [17]. Across the two traits, 44 loci were identified as significantly associated with hearing loss.

Identifying fewer loci with HAID than with HD suggests that HD is a more stringent measure of hearing ability for detecting genetic associations in the UK, at least within the age range examined. The HD analysis may be more highly powered than HAID due to difference in prevalence of positive response for the hearing difficulty question (35% in the white British) being much higher than for the wearing a HAID question (5.2%) [17]; as prevalence tends toward 50% the power is expected to increase, assuming an underlying normal liability distribution. As a result of the younger age range of the UKB sample (mean 59 years, range of 40–73, in the GWAS sample), relatively few people wear HAIDs. Another difficulty with the HAID phenotype is that many people who could benefit from wearing a HAID choose not to do so regularly (required for a positive response), which might introduce noise into the ARHI phenotype. Presumably, a subject’s response to whether they have HD is a better indicator of their hearing status, particularly for this relatively young sample.

The polygenic risk score (PRS) provides a way of predicting likelihood of developing a trait based on results obtained from large GWAS [19]. Rather than simply considering the significant loci from the study which provides limited power, a PRS may predict a phenotype in a different sample using any number of SNPs, with the effect weights obtained from the base sample GWAS. If it can be demonstrated that significant prediction of a phenotype in a target sample is possible, then the PRS may provide a useful screening test in, say, young adults and allow targeted intervention in the future.

The present study aimed to (1) estimate heritability and environmental contribution of liability to ARHI in multiple samples using different objective and subjective measures of hearing and examine their degree of overlap and (2) examine the extent to which PRS from the recent UKB GWAS is predictive of hearing loss in other samples, both ethnically similar and different, and tested with various different measures of hearing ability. To achieve these aims, we used (1) an ethnically similar sample of twins from the TwinsUK Bioresource who have the questionnaire measures analogous to the UKB HAID and HD measures, as well as hearing ability measured by objective tests and (2) European and non-European (Africans, South-East Asians who are mostly Chinese, and non-Chinese South Asians) samples from the UKB that were not part of the original GWAS.

## Materials and methods

### Samples and genotyping

The UKB comprises 487,401 individuals, the majority of whom are ethnic Northern European (408,254). Multiple additional populations are also present in the UKB, as defined by genetic PCs analysis, including individuals of non-northern European origin (*n* = 52,936 individuals), African origin (*n* = 938), South-East Asian (predominantly Chinese) origin (*n* = 2548) and non-Chinese South Asian origin (*n* = 10,997). Samples were genotyped using one of two custom and highly similar Affymetrix SNP arrays and imputed to the 1000 Genomes reference panel, yielding 9,740,198 SNPs in total. Further details are presented elsewhere [17].

TwinsUK is a large prospective Northern European twin cohort [20] comprising 5654 genotyped individuals. PCs analysis of genotype data was used to confirm ancestry and discard outliers, so the samples included were relatively genetically homogeneous. For the PRS, we included one member of each monozygotic (MZ) twin pair and both members of each dizygotic (DZ) pair, with adjustment for relatedness via simulation. All twins were genotyped on either the HumanHap300 BeadChip or the HumanHap610 QuadChip (Illumina, San Diego, CA, USA).

### Phenotypes

Large-scale assessment of hearing loss via questionnaire is not a new approach [21–23]. For UKB samples, we used three questions addressing ARHI: ‘Do you use a hearing aid most of the time?’ (HAID). Here, participants that responded ‘Yes’ were defined as cases and those that responded ‘No’ were defined as controls. For HD, participants that responded ‘Yes’ to both ‘Do you have any difficulty with your hearing?’ and ‘Do you find it difficult to follow a conversation if there is background noise (such as TV, radio, children playing)?’ were assigned as cases and participants that responded ‘No’ to both questions were assigned as controls. Participants that selected any other combinations of responses were excluded. In addition, individuals that had responded ‘Yes’ to ‘I am completely deaf’ were excluded, as were any controls aged < 50 years.

TwinsUK also employed analogous questions and in addition performed objective tests of hearing ability which yielded two quantitative measures, the web-based speech-in-noise ratio (SNR) derived from the triple digit test and the laboratory-administered pure-tone audiogram (PTA), both previously described [8]. In the TwinsUK sample, HD cases were defined as responding either ‘Yes, diagnosed by doctor or health professional’ or ‘Yes, not diagnosed by health professional’ to ‘Do you suffer from hearing loss?’ while participants that responded ‘No’ were assigned as controls. For TwinsUK HAID, cases responded ‘Yes’ to either ‘Do you wear a hearing aid?’ or ‘Wearing a hearing aid’ while controls responded ‘No’. All twins aged < 40 years were excluded from analysis and if a participant had responded to the question twice (due to the longitudinal nature of the study) the second response was used for analysis. If however the second response indicated that hearing ability had improved, the participant was excluded.

Table 1 contains the number of people affected or unaffected with genotypes in the studied samples, or the number of available genotyped samples for quantitative phenotypes in TwinsUK, age at testing, and percentage of males in the samples. For the heritability analysis of HD, 5316 individuals in sibships were available (2979 MZ), 618 of which were male, and prevalence of HD was 26.7%. For HAID, 4341 individuals with siblings tested were available (2198 MZ), 401 of whom were male, and the trait prevalence = 7.7%. For PTA, 1020 individuals (two males; 460 MZ) were included, while for SNR 1744 individuals (212 males; 1035 MZ) were analysed. The sample prevalence for UKB EA and SA populations were quite similar to those for UKB white British, as was the TwinsUK sample prevalence, but those for AA and CH were markedly lower for both HD and HAID.

**Table 1:** Samples and numbers of individuals for polygenic risk score analyses.

Target sample	Target phenotype	Case	Control	% affected	Mean age	Age SD	Age range	% male
UKB EA	HD	10,491	19,091	35.46	59.13	6.25	40–73	43.9
	HAID	1410	32,656	4.14	56.31	8.18	40–73	46.2
UKB AA	HD	555	2608	17.55	57.50	6.37	40–72	40.9
	HAID	74	5439	1.34	52.25	8.13	39–72	41.0
UKB CH	HD	230	722	24.16	57.31	5.97	40–73	32.4
	HAID	37	1644	2.20	53.00	7.89	40–73	31.8
UKB SA	HD	1592	3180	33.36	58.19	6.54	40–73	52.0
	HAID	263	8127	3.13	54.01	8.49	40–73	51.7
TwinsUK	HD	970	2666	26.68	60.34	10.18	40–89	8.5
	HAID	216	2696	7.42	59.32	9.69	40–87	8.0
	SNR	1092	N/A	N/A	56.83	11.35	21–83	8.7
	PTA	817	N/A	N/A	61.37	8.85	32–86	0.0

UK Biobank data included the following samples: UKB EA—European origin, UKB AA—African origin, UKB CH—South-East Asian (predominantly Chinese) origin and UKB SA—non-Chinese South Asian origin. Target phenotypes are hearing difficulties (HD) and wearing a hearing aid (HAID).

### Statistical analysis

#### Heritability estimation

We employed the MAN package [24] to perform a quasi-variance components joint maximum likelihood analysis to fit the liability transmission and estimate the components of variance: additive genetic (V_AD_), shared environment (V_SB_), and non-shared or random environment (V_RS_), along with the affection threshold τ on a liability scale. MAN was also used to estimate variance components for quantitative traits and covariance between traits. These parameters were allowed to vary by sex and the linear effect of age on the phenotypes was simultaneously modelled and tested. This was performed for the TwinsUK and the UKB samples. For the UKB first-degree relatives and MZ twins were identified by estimating kinship matrix among all individuals and selecting those with kinship coefficients > 0.495—suggesting MZ twins—and >0.25 implying first-degree relatives. Overall, 3275 individuals with phenotypes had first-degree relatives and of those 70 were members of an MZ pair.

#### PRS analysis

PRS analysis was performed using the PRSice-2 package [19, 25]. Briefly, the approach constructs a polygenic score by summing all trait-associated alleles in a target sample, weighted by the effect size of each allele in a base GWAS. SNPs in linkage disequilibrium (LD) are grouped together so as not to give extra weight to a single marker. PRS are computed at various thresholds of *p* value significance in the base GWAS and the optimal threshold, in terms of maximum variance explained in the target sample, was estimated. The significance test of that optimal *p* value was adjusted by permutation (*N* = 10,000) to correct for the effective number of cutoffs examined as well as underlying correlated errors due to familial relationships (important for the twin sample, where we included both members of each DZ pair). We used PRSice-2 to predict the four hearing measures in the TwinsUK sample and two hearing measures in the four UKB subsamples from weights derived from the two GWAS performed in the UKB on the ethnic British sample [17]. We filtered out imputed base SNPs with information scores < 0.9, target SNPs with genotype missingness > 0.05, MAF < 0.01 and information scores < 0.9. All SNPs within 250 kb and with an *r*^2^ > 0.1 with the target were clumped to adjust for LD in the target population. A SNP was considered part of a region of clumped SNPs if it had an *r*^2^ > 0.8 with any SNP in that region. For TwinsUK, we analysed one member of each MZ twin pair and both members of DZ twin pairs. After removing duplicate and ambiguous variants from the base GWAS, 5,416,754 SNPs were available in the TwinsUK sample, which were also present in the base sample and survived filtering. For the UKB samples, 8,724,365 SNPs were included for the AA sample; 4,731,237 for CH; 7,399,812 for EA; and 5,711,698 for SA. Numbers of SNPs available after LD clumping are presented in Supplementary Table S1.

#### GCTA-GREML analysis

We estimated heritability of HAID and HD in TwinsUK using the genome-wide GCTA-GREML approach [26–29]. Because DZ twin pairs are related we partitioned the familial variance into that due to sibship or close familial relationship (using semi-arbitrary cut-off of genetic relatedness > 0.05) and that due to SNPs. Estimates were computed separately by chromosome, with SNP genetic variance summed across chromosomes, as well as computed genome-wide.

## Results

### Familial correlations and variance components estimates in TwinsUK

Significant genetic variation for each of the ARHI phenotypes was detected in both samples, independent of age and sex covariates. For HD in the TwinsUK sample, the contribution of additive genetic variance to the total liability was 50.9% (as estimated from the most parsimonious model), with heritability (covariates variance not in the denominator) of 55.7% (*χ*^2^ = 22.8, *p* < 1.8 × 10^–6^ vs model assuming no additive genetic effect). In addition, age and sex made a statistically significant contribution to HD liability variation (7.7% was explained by age differences, *χ*^2^ = 179.1, *p* = 7.6 × 10^–41^, and 0.97% of variation was attributable to sex, *χ*^2^ = 24.62, *p* < 7 × 10^–7^). For HAID in TwinsUK, sex differences exerted no effect, but age accounted for 26.17% of the variance in liability (*χ*^2^ = 235.61, *p* < 10^–52^). The contribution of the additive genetic component was impressive: 52.6%, with heritability equal to 71.2% (*χ*^2^ = 10.59, *p* < 0.002). Shared environment was estimated to be zero for both phenotypes (see Supplementary Table S2 for further details). The objective measures of hearing in TwinsUK, SNR and PTA, were subjected to lognormal and 4th root transformations, respectively, to achieve normality prior to variance components analysis. Heritability was estimated for PTA to be 67.9% (*χ*^2^ = 32.48, *p* < 10^–7^), but for SNR only 19.7% and not quite statistically significant (*χ*^2^ = 3.53, *p* = 0.06). Shared environmental variance was again estimated at 0 for both variables (see Supplementary Table S2).

### Bivariate relationships of objective measures and self-reported hearing in TwinsUK

Bivariate analyses revealed significant genetic correlations between and among self-reported hearing loss and objective measures on PTA and SNR, ranging from 0.30 ± 0.11 (HD/SNR) to 0.86 ± 0.07 (HD/HAID, see Supplementary Table S3 for details), with non-shared environmental correlations ranging from 0.36 ± 0.11 (SNR/PTA) to 0.75 ± 0.13 (HAID/PTA). All these correlations were statistically significant (see Supplementary Table S3). Of special interest in this context were the high genetic correlations between the questionnaire-assessed phenotypes and the PTA, validating the use of questionnaires in genetic association studies of ARHI.

### Variance components estimates from UK Biobank

In the UKB sample, the proportion of MZ twins was very small (2.4%), so clear-cut discrimination between the shared environment and additive genetic components was not possible. However, the 2-df tests for heritability plus shared environmental variance were significant for both HD (*p* < 0.0033) and HAID (*p* < 0.0034), implying significant familial correlation in the sample: whether the source is genetic or shared environmental factors cannot be distinguished. Assuming that as in the TwinsUK sample the major factor is genetic, constraining the shared environment to 0 in the most parsimonious models produced a significant estimate of heritability in HD (0.317 ± 0.097), with age and sex explaining 7.02% of liability variation. Similarly, for HAID heritability was estimated to be 0.646 ± 0.217, with age and sex explaining 8.71% of the variance.

### PRS generated in UK Biobank—self reported hearing loss

**Fig. 1:**
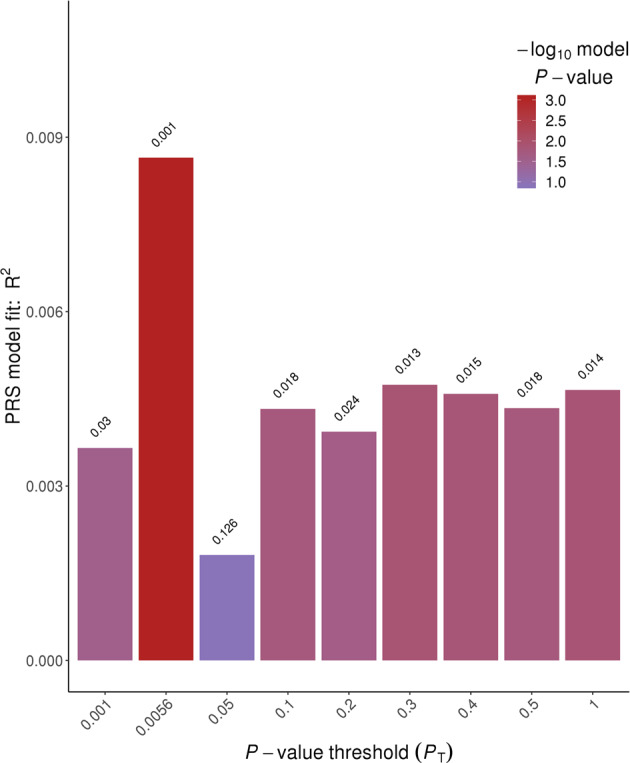
Hearing aid (HAID) polygenic risk score (PRS) from UK Biobank British genome-wide association study used to predict HAID in TwinsUK. Total variance explained by the PRS for multiple *p* value thresholds for inclusion of SNPs, with the red bar indicating the optimal *p* value threshold, explaining the maximum amount of variance in HAID in the target sample. See text for further details.

**Fig. 2:**
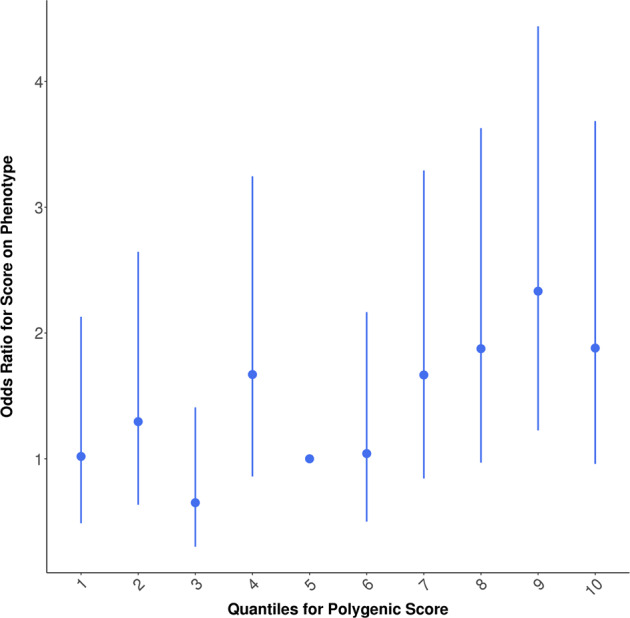
Odds ratios for ten evenly spaced quantiles of polygenic risk score (PRS) for the optimal *p* value threshold, for hearing aid (HAID) from UK Biobank British genome-wide association study used to predict HAID in TwinsUK. See text for further details.

**Fig. 3:**
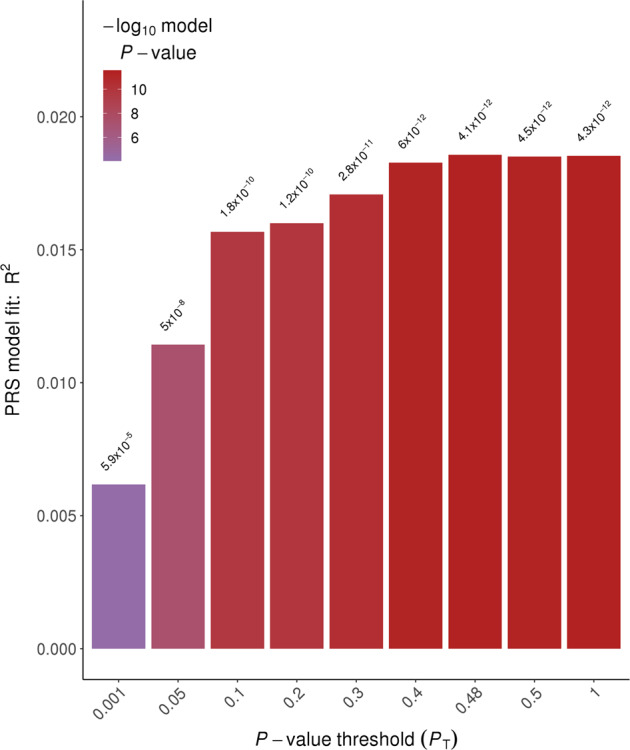
Hearing difficulties (HD) polygenic risk score (PRS) from UK Biobank British genome-wide association study used to predict HD in TwinsUK. Total variance explained by the PRS for multiple *p* value thresholds for inclusion of SNPs, with the highest bar indicating the optimal *p* value threshold, explaining the maximum amount of variance in HD in the target sample. See text for further details.

**Fig. 4:**
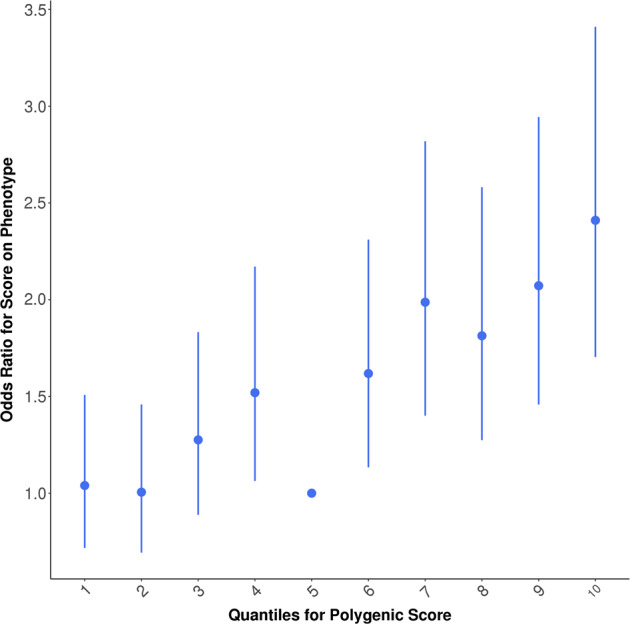
Odds ratios for ten evenly spaced quantiles of polygenic risk score (PRS) for the optimal *p* value threshold, for hearing difficulties (HD) from UK Biobank British genome-wide association study used to predict HD in TwinsUK. See text for further details.

In general, PRS generated from the UKB GWAS was predictive of TwinsUK hearing phenotypes (see Table 2). We also performed mixed model regressions to account for familial resemblance within DZ twin pairs and present the *p* values in Table 2, noting that *p* values are similar though on average slightly less significant, with effect sizes also very similar. HAID PRS significantly predicted HAID in TwinsUK (empirical *p* < 0.011; Fig. 1). In Fig. 2, we present odds ratios for 10 evenly spaced quantiles of PRS, using the optimal threshold of SNP selection from PRSice, and can see the increasing odds ratios rising through the quantiles for HAID. Figure 3 represents HD PRS from UKB significantly predicting HD in the twins (*p* = 0.0001). The odds ratios presented in the corresponding quantile plot in Fig. 4 shows prediction is more robust for HD than for HAID. Cross-phenotype prediction showed that HAID did not significantly predict HD (*p* = 0.146; Fig. S1), but HD PRS significantly predicted HAID in the twins (*p* = 0.0022; Fig. S2). Despite the statistical significance of the predictions, variance explained by the PRS was uniformly small, just under 2% for the best prediction (HD on HD). Finally, HD PRS significantly predicted PTA in TwinsUK, with HAID also being predictive of PTA, though the *p* value was just < 0.05.

**Table 2:** PRSice-2 polygenic risk score (PRS) prediction results for TwinsUK target sample, for optimal *p* value threshold.

Measure pair	Threshold	PRS *R*^2^	Full *R*^2^	Null *R*^2^	Coefficient	Standard error	*p*	*p*_mixed_	No. of SNP	Empirical *p*
HAID–HAID	0.0056	0.0086	0.1923	0.1836	10,395.2	3121.25	0.0008	0.0012	4266	0.0109
HAID–HD	0.00025	0.0022	0.0748	0.0726	958.2	398.569	0.0162	0.0168	377	0.1461
HAID–PTA	0.00055	0.0074	0.3233	0.3159	26,501.5	8898.04	0.0030	0.0119	712	0.0377
HAID–SNR	0.00055	0.0007	0.1114	0.1107	1824.8	1967.61	0.3539	0.396	712	0.9737
HD–HAID	0.12	0.0113	0.1950	0.1836	21,895.5	5754.99	0.00014	0.00022	46,877	0.00228
HD–HD	0.48	0.0186	0.0911	0.0726	36,885.8	5318.89	4.066E−12	5.23E−11	100,190	<0.0001
HD–PTA	0.37	0.0182	0.3341	0.3159	36,4042	77,141.3	2.786E−06	3.36E−06	88,278	<0.0001
HD–SNR	0.14005	0.0047	0.1153	0.1107	25,480.3	10,639.8	0.0168	0.0281	51,433	0.1122

Measure pair denotes base sample polygenic risk scores constructed from the UK Biobank genome-wide association study on British samples of either wearing a hearing aid (HAID) or having hearing difficulties (HD), respectively, predicting the TwinsUK target phenotypes HAID, HD, pure-tone audiogram (PTA), and signal-in-noise (SNR), respectively. Threshold is the *p* value threshold for SNP inclusion that maximised variance explained in the respective target phenotype. PRS *R*^2^ is the variance in the target phenotype explained by the PRS. Full *R*^2^ is the variance explained by the full-model regression, which includes the PRS as well as the covariates age and sex (no sex covariate for PTA, since there were no males in that sample), while Null *R*^2^ does not include the PRS, just the covariates. Coefficient is the regression coefficient for the PRS term, with Standard Error and *p* its corresponding standard error and *p* value. *P*_mixed_ was obtained from running a mixed model accounting for correlation among dizygotic twin pairs. No. of SNP is the number of SNPs included in the PRS and empirical *p* is the *p* value obtained from simulation, which corrects for both multiple thresholds tested in order to obtain the optimal threshold and for relatedness in the sample, such as the inclusion of DZ twin pairs.

### PRS generated in UK Biobank—from self-report to objective measures of hearing

We also examined whether the UKB PRS could predict the objective quantitative measures of hearing available, SNR and PTA. Neither HAID nor HD significantly predicted SNR, but HD significantly predicted PTA (*r*^2^ = 0.06, *p* = 0.03), despite the small sample size. Note here that the estimate of the SNR heritability was not significant (see above).

The PRS computed from UKB British samples were generally not predictive of the non-European subsamples (Table 3). Specifically, neither HAID nor HD significantly predicted HAID or HD in the Chinese and African subsamples. However, for South Asian, HD PRS significantly predicted HD. While variance explained was small, the two PRS did significantly predict both phenotypes in the European samples (see prediction as a function of *p* value threshold in Figs, S3–6). Looking at the magnitude of prediction another way, for the European target sample, in terms of odds ratios, the top 1% of HD PRS scorers were 1.77 times more likely to have HD than the bottom 99%, while the top 5% were 1.55 times more like to be affected.

**Table 3:** PRSice-2 polygenic risk score (PRS) prediction results for UK Biobank target samples, for optimal *p* value threshold.

Measure pair	Target sample	Threshold	PRS *R*^2^	Full *R*^2^	Null *R*^2^	Coefficient	Standard error	*p*	No. of SNP	Empirical *p*
HAID–HAID	AA	0.36	0.0104	0.0248	0.0144	112,697	40,950.5	0.0059	132,397	0.0701
HAID–HD	AA	0.017	0.0033	0.0991	0.0958	−7664.44	2951.83	0.0094	11,338	1
HD–HAID	AA	0.016	0.0015	0.0159	0.0144	4257.94	4092.96	0.2982	15,955	1
HD–HD	AA	0.019	0.0006	0.0964	0.0958	−2025.13	1827.21	0.2677	17,932	1
HAID–HAID	CH	0.14005	0.0041	0.0212	0.0172	31,395.8	27,561.6	0.2547	42,704	1
HAID–HD	CH	0.0003	0.0025	0.0687	0.0662	−730.731	569.454	0.1994	317	1
HD–HAID	CH	0.0091	0.0210	0.0382	0.0172	10,772.2	4188.19	0.0101	7853	0.1025
HD–HD	CH	0.015	0.0095	0.0757	0.0662	5924.37	2355.89	0.0119	11,034	0.1085
HAID–HAID	EA	0.0092	0.0017	0.0701	0.0684	15,670.8	3775.81	3.320E−05	10,648	0.0009
HAID–HD	EA	0.35	0.0013	0.0468	0.0455	53,336.3	10,047.5	1.106E−07	183,790	<0.0001
HD–HAID	EA	0.019	0.0027	0.0711	0.0684	13207	2506.83	1.376E−07	24,107	<0.0001
HD–HD	EA	0.018	0.0124	0.0579	0.0455	18,389.9	1111.69	1.821E−61	23,187	<0.0001
HAID–HAID	SA	0.045	0.0032	0.0424	0.0392	−19,437.9	7590.78	0.0104	28,737	1
HAID–HD	SA	0.1	0.0007	0.0457	0.0450	−9085.58	5864.3	0.1213	55,832	1
HD–HAID	SA	0.0037	0.0014	0.0407	0.0392	−2137.48	1241.07	0.0850	5932	1
HD–HD	SA	0.28005	0.0075	0.0525	0.0450	24,413.7	4746.59	2.698E−07	123,566	<0.0001

Measure pair denotes base sample polygenic risk scores constructed from UK Biobank genome-wide association study on British samples of either wearing a hearing aid (HAID) or having hearing difficulties (HD), respectively, predicting the UK Biobank target phenotypes HAID and HD, respectively. Target sample refers to the four UK Biobank subpopulations, African (AA), Chinese (CH), European (EA), and South Asian (SA), described in the text. Threshold is the *p* value threshold for SNP inclusion that maximised variance explained in the respective target phenotype. PRS *R*^2^ is the variance in the target phenotype explained by the PRS. Full *R*^2^ is the variance explained by the full-model regression, which includes the PRS as well as the covariates age and sex, while Null *R*^2^ does not include the PRS, just the covariates. Coefficient is the regression coefficient for the PRS term, with standard error and p its corresponding standard error and *p* value. No. of SNP is the number of SNPs included in the PRS and empirical *p* is the *p* value obtained from simulation, which corrects for both multiple thresholds tested in order to obtain the optimal threshold and for relatedness in the sample, such as the inclusion of DZ twin pairs.

### GCTA–GREML in TwinsUK

In TwinsUK, we estimated SNP-based and familial heritability with GCTA’s GREML using one member of each MZ twin pair and both members of each DZ twin pair. SNP-based heritability was 0.00 for HD and 0.05 for HAID. Additional variance explained by the DZ familial relationship was 0.34 for HD and 0.37 for HAID. Very little of the DZ correlation was explained by the SNP heritability, consistent with the PRS results. We performed this GREML analysis separately by chromosome as well, with results shown in Table 4. Consistent with the genome-wide GREML, SNP heritability was higher for HAID than for HD, though the sums of the heritabilities across chromosomes were greater than what was found for the single genome-wide analysis. However, the absolute difference between HAID and HD heritabilities was almost the same (0.05). This inflation of individual chromosome heritabilities is likely a result of population structure inflating the estimates.

**Table 4:** GCTA SNP heritability estimates by chromosome, in TwinsUK sample, for hearing aid (HAID) and hearing difficulties (HD) phenotypes.

	HAID			HD		
Chromosome	*h*^2^-SNP	SE	*p*	*h*^2^-SNP	SE	*p*
1	0.0044	0.0315	4.45E−01	1E−06	0.0245	5.00E−01
2	1E−06	0.0322	5.00E−01	1E−06	0.0254	5.00E−01
3	0.0084	0.0281	3.82E−01	0.0145	0.0231	2.64E−01
4	0.0014	0.0281	4.81E−01	0.0072	0.0223	3.72E−01
5	0.0333	0.0283	1.09E−01	1E−06	0.0213	5.00E−01
6	0.0037	0.0193	4.14E−01	0.0126	0.0192	2.52E−01
7	1E−06	0.0262	5.00E−01	1E−06	0.0216	5.00E−01
8	1E−06	0.0252	5.00E−01	1E−06	0.0185	5.00E−01
9	1E−06	0.0239	5.00E−01	1E−06	0.0217	5.00E−01
10	0.0068	0.0238	3.84E−01	0.0071	0.0194	3.55E−01
11	0.0341	0.0233	3.39E−02	0.0093	0.0160	2.58E−01
12	0.0134	0.0242	2.80E−01	1E−06	0.0188	5.00E−01
13	1E−06	0.0224	5.00E−01	0.0066	0.0169	3.42E−01
14	0.0119	0.0217	2.96E−01	1E−06	0.0167	5.00E−01
15	0.0044	0.0195	4.09E−01	1E−06	0.0166	5.00E−01
16	1E−06	0.0228	5.00E−01	1E−06	0.0176	5.00E−01
17	1E−06	0.0157	5.00E−01	0.0042	0.0132	3.57E−01
18	1E−06	0.0212	5.00E−01	0.0128	0.0178	2.35E−01
19	0.0136	0.0207	2.69E−01	1E−06	0.0153	5.00E−01
20	0.0044	0.0191	4.07E−01	0.0049	0.0160	3.82E−01
21	1E−06	0.0169	5.00E−01	1E−06	0.0137	5.00E−01
22	1E−06	0.0155	5.00E−01	1E−06	0.0121	5.00E−01
Sum	0.14			0.08		

*h*^2^-SNP is the proportion of variance explained by all common SNPs on the chromosome, with SE and *p* being the standard error of the estimate of chromosome heritability and its corresponding *p* value.

## Discussion

We have previously demonstrated that the SNR test shares considerable genetic overlap with PTA, making it suitable for large scale association studies of hearing [8]. This next piece of work on hearing phenotypes was performed to ascertain the usefulness to genetic studies of responses to questions about hearing difficulty and HAID use. If simple, quick questionnaires provide a suitable phenotype for genetic studies then huge potential will be unlocked cheaply and easily by applying such hearing questions to existing bioresources around the world. Advances in understanding this important age-related phenotype and cause of disability could follow rapidly. We began by examining the heritability of ARHI in TwinsUK using both a classical twin study [8] and a SNP-based genome-wide approach, and doing similar in the very much larger UKB sample, by extracting first-degree relatives and MZ twins.

The phenotypes of interest have been demonstrated to have modest to moderate heritability, depending on method of estimation and sample used. The low estimate of heritability found in the UKB sample using MAN variance components analysis likely results from the very small number of MZ twin pairs available. However, recently, we reported SNP-based estimates of heritability using BOLT-LMM for these UKB traits, with HD heritability estimated to be 11.7% (SE = 0.1%) and HAID to be 2.9% (SE = 0.1%), and a bit higher using the liability scale (19% and 13%, respectively) [17]. In the twin sample we found substantial heritability of all four phenotypes—objective and self-report measures—related to ARHI. This was expected because all forms of heritability are considered in twin studies, while only that transmitted by common variants is captured by SNP-based methods. Thus the findings in UKB are in agreement with those from twins, and demonstrate that questionnaire responses provide a good surrogate for identifying genetic biomarkers in ARHI. That HAID prescription in the UK relies on having demonstrated an abnormal PTA perhaps implies it is a more relevant proxy for the objective measure.

The substantial genetic correlations among the four phenotypes explored in TwinsUK suggests that a simple question of whether or not one experiences hearing difficulty or uses an HAID is a useful proxy for the more elaborate tests of hearing ability. The PTA has long been considered the gold standard measure of hearing but it requires expensive equipment and trained personnel to administer it. Our work further validates the GWAS findings from UKB hearing questions and justifies the use of the simple binary questionnaire responses, lending support to the accepted view that quality of phenotype is less of a concern if a very large sample is available, as demonstrated early on by the personal genomics company 23andMe [30]. They showed that simple questionnaires assessing disease on a large (at the time) sample of 20,000 unselected individuals was sufficient to replicate 75% of published findings on diseases previously obtained on clinical samples and considered replicable.

It is disappointing, but perhaps not surprising, that PRS generated in one population (ethnic Northern European) are not useful predictors of phenotype in ethnically distinct populations. This is possibly due to the small sample size, as well as allele frequency and LD differences between populations. It is generally accepted that cross-population PRS is not a powerful approach and a comprehensive review of PRS published from 2008 to 2017 demonstrated this [31]. Furthermore, a previous attempt to replicate a SNP identified on a hearing loss GWAS of Europeans in a Han-Chinese sample was also unsuccessful [32]. That said, within the same Northern European (TwinsUK) or similar European populations, prediction was robust and we include the other populations for completeness, to add to the literature that cross-population prediction is not particularly useful. However, PRS of one phenotype could predict another hearing phenotype in a target sample of similar genetic ancestry.

Cross-population prediction is likely somewhat affected by power due to small sample size. While the size of the European sample was large, the other population groups had relatively small sample sizes. It is likely that much larger samples are needed to overcome the mismatch in population origin and corresponding differences in allele frequencies. In fact, the estimates of variance explained by PRS in the samples of Asian and African origin were not appreciably different from those found in Europeans, so if the estimates remain the same and a larger sample were available prediction could be significant across populations.

While the proportion of variance explained by the PRS was uniformly small at 2%, despite high trait heritability estimated from familial correlations, such PRS may still be useful for identifying those at risk for hearing loss. For instance, in our European target population, knowing that someone is 1.8 times more at risk than the general population, as is the case for 1% of the population, could have important utility in identifying people at young age who should take extra precaution in exposure to loud noise, for example. With the current trend of large-scale sequencing efforts being undertaken in multiple countries, prediction will only improve and the utility of PRS for predicting ARHI, as with other complex diseases, will increase. In summary, our work demonstrates that questionnaire-based methods to phenotype adults for hearing loss in gene finding studies is robust and paves the way for many existing genotyped bioresources to collect simple information which could revolutionise the study of genetic variants causing ARHI.

## Supplementary information

Supplementary Material
