# Supplementary material for: Self-reported hearing loss questions provide a good measure for genetic studies: a polygenic risk score analysis from UK Biobank
Source: Eur J Hum Genet. 2020 Mar 20;28(8):1056–65. doi: 10.1038/s41431-020-0603-2 (PMC7382483; doi:10.1038/s41431-020-0603-2)
Supplement: Supplementary file 1 — Supplementary Material [file 41431_2020_603_MOESM1_ESM.docx]

Stacey S Cherny, Gregory Livshits, Helena RR Wells, Maxim B Freidin, Ida Malkin, Sally Dawson and Frances MK Williams.

Self-reported hearing loss questions provide a good measure for genetic studies: a polygenic risk score analysis from UK Biobank.

**Supplementary Material**


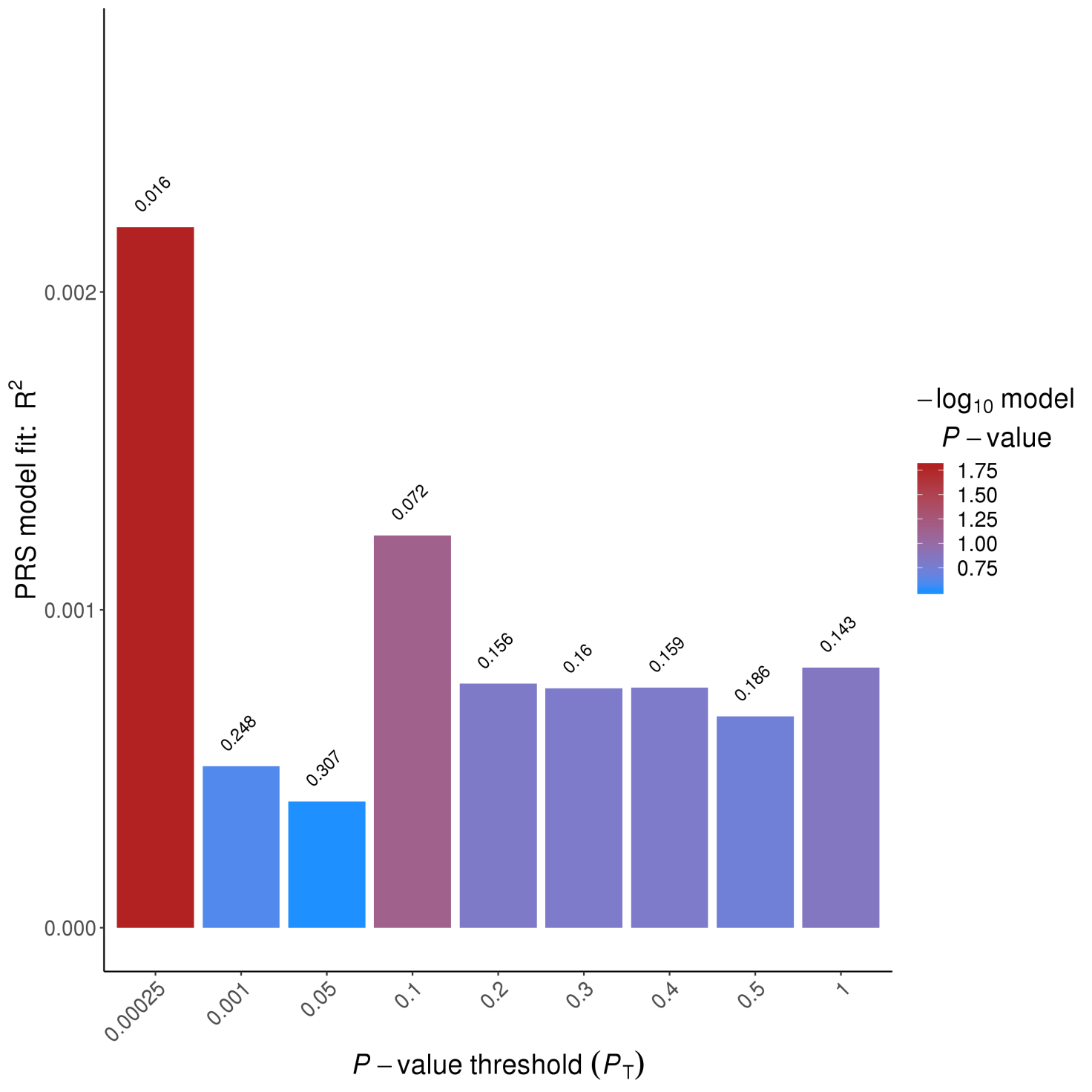
**Figure S1: HAID PRS predicting HD in twins.** Total variance explained by the polygenic risk score (PRS) for multiple p-value thresholds for inclusion of HAID SNPs in the PRS, with the red bars indicating the optimal p-value threshold, explaining the maximum amount of variance in HD in the target sample.


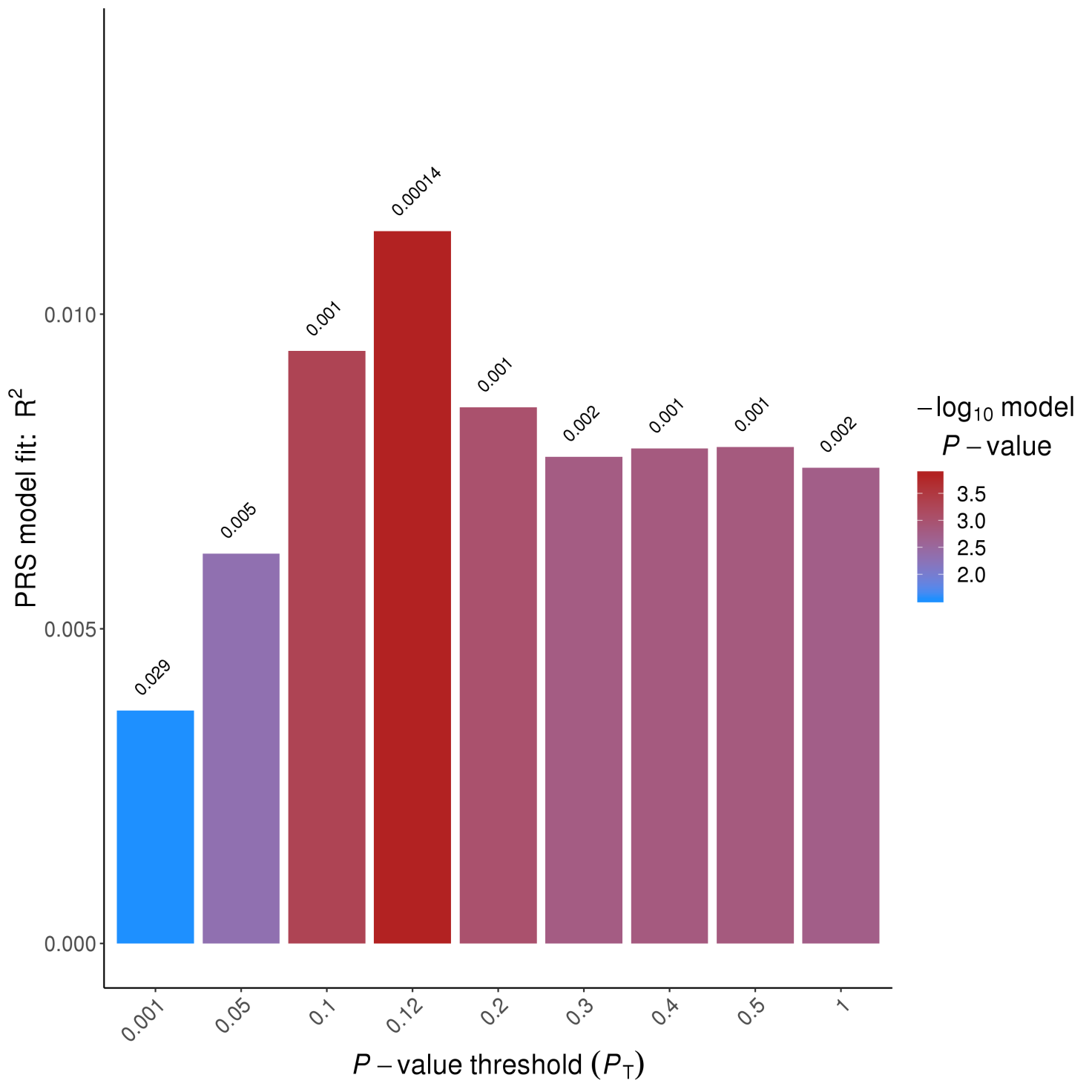
**Figure S2: HD PRS predicting HAID in twins.** Total variance explained by the polygenic risk score (PRS) for multiple p-value thresholds for inclusion of HD SNPs in the PRS, with the red bars indicating the optimal p-value threshold, explaining the maximum amount of variance in HAID in the target sample.

**Figure S3: HAID PRS predicting HAID in EA.** Total variance explained by the polygenic risk score (PRS) for multiple p-value thresholds for inclusion of HAID SNPs in the PRS, with the red bars indicating the optimal p-value threshold, explaining the maximum amount of variance in HAID in the target sample.


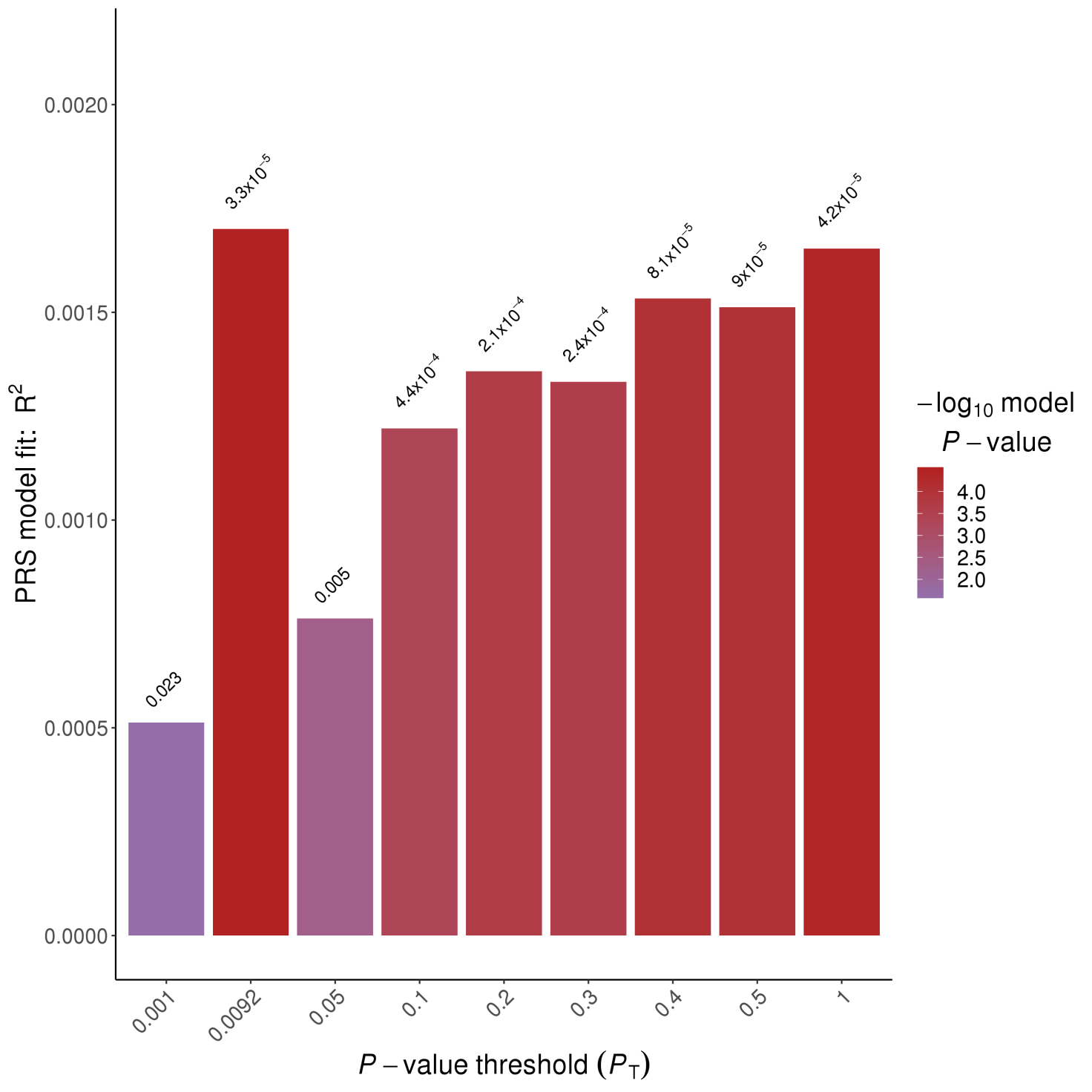


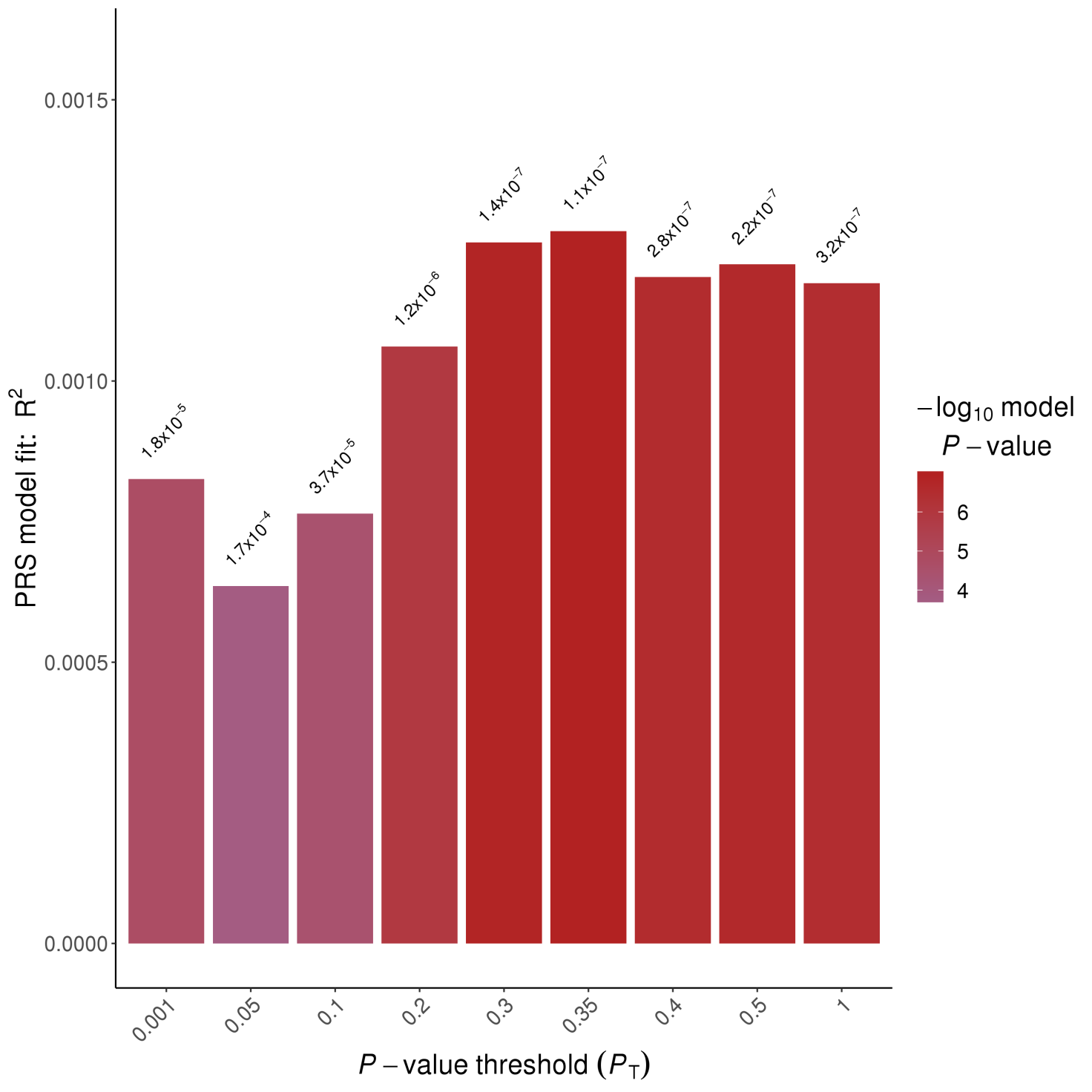
**Figure S4: HAID PRS predicting HD in EA.** Total variance explained by the polygenic risk score (PRS) for multiple p-value thresholds for inclusion of HAID SNPs in the PRS, with the red bars indicating the optimal p-value threshold, explaining the maximum amount of variance in HD in the target sample.


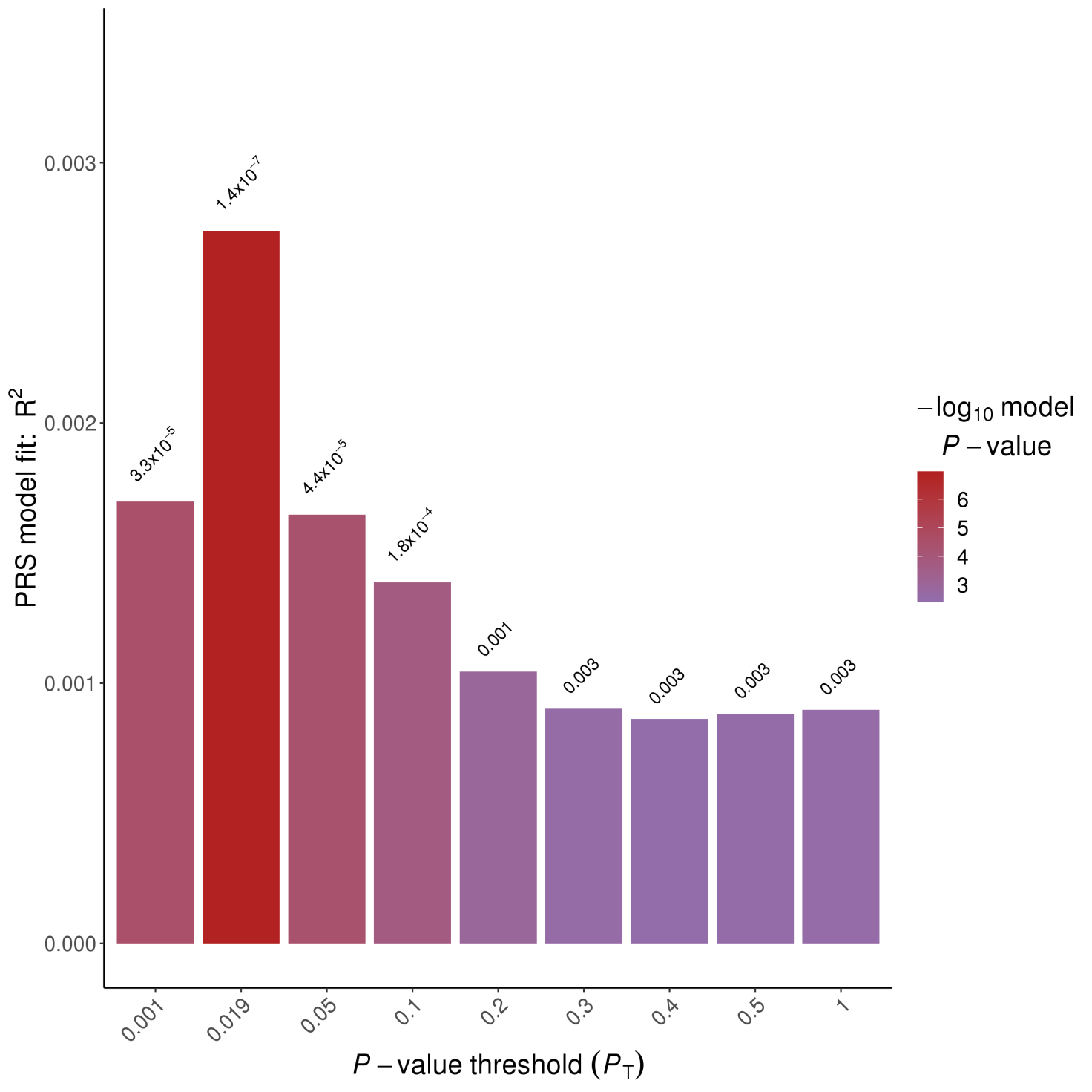
**Figure S5: HD PRS predicting HAID in EA.** Total variance explained by the polygenic risk score (PRS) for multiple p-value thresholds for inclusion of HD SNPs in the PRS, with the red bars indicating the optimal p-value threshold, explaining the maximum amount of variance in HAID in the target sample.


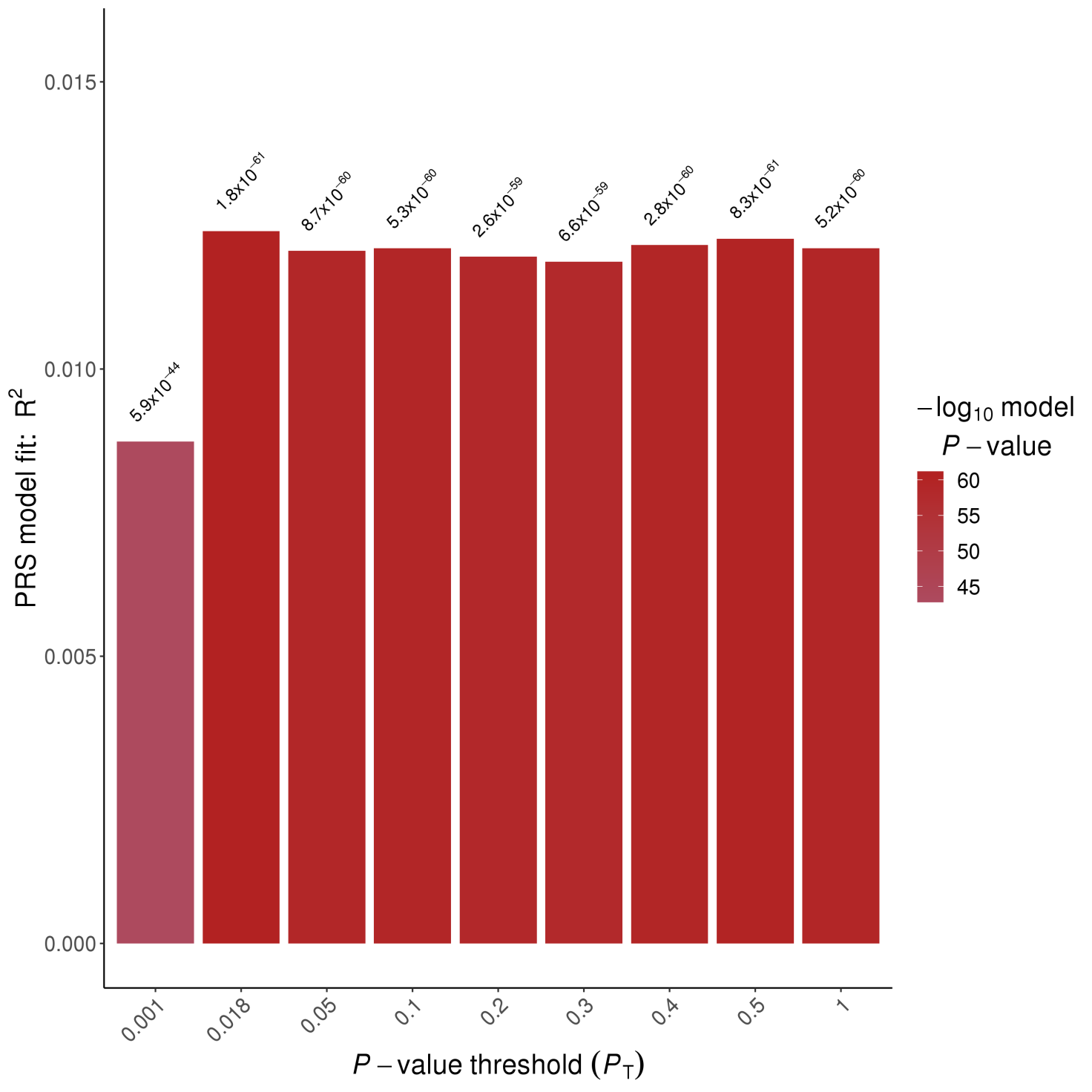
**Figure S6: HD PRS predicting HD in EA.** Total variance explained by the polygenic risk score (PRS) for multiple p-value thresholds for inclusion of HD SNPs in the PRS, with the red bars indicating the optimal p-value threshold, explaining the maximum amount of variance in HD in the target sample.

**Table S1: Number of SNPs available for PRS analysis after LD clumping**

|  | **Base sample (UKBiobank white British)** | |
| --- | --- | --- |
| **Target sample** | **HAID** | **HD** |
| TwinsUK | 130,285 | 130,170 |
| AA | 242,415 | 242,486 |
| CH | 118,670 | 118,528 |
| EA | 319.083 | 318.774 |
| SA | 245,442 | 245,262 |

**Table S2: Contribution of putative genetic factors to liability to HD and HAID binary phenotypes (using quasi-variance components), and variance components analysis (VCA) of quantitative phenotypes PTA and SNR in TwinsUK sample.**

| **Phenotype** | **HD** | | **HAID** | |  | **PTA*** | | **SNR*** | |
| --- | --- | --- | --- | --- | --- | --- | --- | --- | --- |
| **Parameter** | **Free** | **MPM** | **Free** | **MPM** | **Parameter** | **Free** | **MPM** | **Free** | **MPM** |
| **σ_AD_^2^** | 0.5103 | 0.5086 | 0.5202 | 0.5256 | **σ_AD_^2^** | 0.6788 | 0.6793 | 0.1970 | 0.1970 |
| **σ_SB_^2^** | 0 | (F) 0 | 0 | (F) 0 | **σ_SB_^2^** | 0.0000 | (F) 0 | 0 | (F) 0 |
| **σ_RS_^2^(C)** | 0.4068 | 0.4050 | 0.2150 | 0.2127 | **σ_RS_^2^** | 0.3212 | 0.3207 | 0.8030 | 0.8030 |
| **L_AF_** | 0.2666 | 0.2666 | 0.0773 | 0.0773 |  |  |  |  |  |
| **α_0m_** (C) | 0.2563 | 0.2625 | 0.1766 | -0.0392 | **μ_m_** | -0.8072 | -0.0017 | 0.2143 | 0.2143 |
| **α_0f_** | -0.0347 | -0.0356 | -0.0593 | (=**α_0m_**) -0.0392 | **μ_f_** | -0.0003 | (=**μ_m_**) -0.0017 | -0.0572 | -0.0572 |
| **β _Age m_** | 0.2751 | 0.2806 | 0.5196 | 0.5228 |  |  |  |  |  |
| **β _Age f_** | 0.2751 | (=**β_Age m_**) 0.2806 | 0.5196 | (=**β_Age m_**) 0.5228 |  |  |  |  |  |
| **L_Lab_** | 0.6312 | 0.6192 | 1.4178 | 1.4310 |  |  |  |  |  |
| **LH** | -2845.72 | -2845.74 | -729.905 | -732.018 | **LH** | -1292.71 | -1293.09 | -1268.57 | -1268.57 |
| χ^2^ |  | 0.02 |  | 4.23 | χ^2^ |  | 0.76 |  | 0 |
| P |  | 0.9804; 2df |  | 0.2381; 3df | P |  | 0.6839; 2df |  | 1 |

* To achieve normality, PTA* was transformed as 4^th^ root(PTA), SNR* transformed by LN(14+SNR), with both adjusted for age (power 2 polynomial) and standardized. σ_AD_^2^, σ_SB_^2^, σ_RS_^2^ are variance components attributable to additive genetic, common twin (sib), and random environment factors, respectively; L_AF_ & L_Lab_ are population prevalence and threshold for liability score, respectively; α_0f_ & β _Age_  are linear regression coefficients for age.

**Table S3. Matrix of genetic and environmental correlations among variables in TwinsUK.** Diagonal elements are additive genetic components as estimated from the most parsimonious model. R_G_ and R_E_ are the respective genetic and environmental correlations with their respective asymptotic standard errors. LRT_G_ and LRT_E_ are likelihood ratio tests vs a model assuming the corresponding parameter is fixed to zero.

| **Variable** | **HD** | **HAID** | **PTA** | **SNR** |
| --- | --- | --- | --- | --- |
| **HD** | 0.5086 ± 0.1069 |  |  |  |
| **LRT** | χ^2^=22.0, p<10^-5^ |  |  |  |
|  |  |  |  |  |
| **HAID** |  | 0.5256 ± 0.1682 |  |  |
| **LRT** |  | χ^2^=10.5, p<0.002 |  |  |
| **R_G_** | 0.8594 ± 0.0667 |  |  |  |
| **R_E_** | 0.6775 ± 0.0806 |  |  |  |
| **LRT_G_** | χ^2^=166.2, p<10^-36^ |  |  |  |
| **LRT_E_** | χ^2^=70.6, p<10^-15^ |  |  |  |
|  |  |  |  |  |
| **PTA** |  |  | 0.6793 ± 0.1192 |  |
| **LRT** |  |  | χ^2^=32.5, p<10^-7^ |  |
| **R_G_** | 0.6659 ± 0.0821 | 0.8364 ± 0.0995 |  |  |
| **R_E_** | 0.5459 ± 0.0849 | 0.7497 ± 0.1344 |  |  |
| **LRT_G_** | χ^2^=65.7, p<10^-14^ | χ^2^=70.7, p<10^-15^ |  |  |
| **LRT_E_** | χ^2^=41.3, p<10^-9^ | χ^2^=31.1, p<10^-7^ |  |  |
|  |  |  |  |  |
| **SNR** |  |  |  | 0.1970 ± 0.0921 |
| **LRT** |  |  |  | χ^2^=3.52, p= 0.06 |
| **R_G_** | 0.2995 ± 0.1075 | 0.8231 ± 0.1535 | 0.4567 ± 0.1603 |  |
| **R_E_** | 0.3802 ± 0.0669 | 0.4925 ± 0.1057 | 0.3642 ± 0.1098 |  |
| **LRT_G_** | χ^2^=7.76, p<.03 | χ^2^=28.8, p<6×10^-7^ | χ^2^=8.12, p<0.005 |  |
| **LRT_E_** | χ^2^=32.3, p<10^-6^ | χ^2^=21.7, p<10^-5^ | χ^2^=11.0, p<0.001 |  |
